# Integrated Impact of Post-TAVR Cardiac Damage and Pacemaker Implantation on Long-Term Outcomes

**DOI:** 10.3390/biomedicines14071569

**Published:** 2026-07-13

**Authors:** Xinyue Yang, Ruisi Tang, Yijun Yao, Fei Chen, Xingzhou Pu, Xi Wang, Jianyong Wang, Chengqiang Liao, Yun Bao, Chao Li, Yiming Li, Mao Chen

**Affiliations:** 1 Department of Cardiology, West China Hospital, Sichuan University, No. 37 Guoxue Street, Chengdu 610041, China; 2 Laboratory of Cardiac Structure and Function, Institute of Cardiovascular Diseases, West China Hospital, Sichuan University, Chengdu 610041, China; 3 College of Computer Science, Sichuan University, Chengdu 610040, China

**Keywords:** aortic stenosis, cardiac damage staging classification, permanent pacemaker, transcatheter aortic valve replacement

## Abstract

**Background/Objectives**: Post-procedural permanent pacemaker implantation (PPMI) and cardiac damage are individually associated with adverse outcomes following transcatheter aortic valve replacement (TAVR). However, their joint impact has not been systematically evaluated. **Methods**: Individuals who underwent TAVR procedures between 2013 and 2024 were retrospectively enrolled and classified into four groups based on their PPMI and cardiac damage status. Univariate and multivariable Cox regression were used to analyze the association of these factors with long-term outcomes, while receiver operating characteristic (ROC) curves, decision curve analysis (DCA), net reclassification improvement (NRI), and integrated discrimination improvement (IDI) were used to further assess their predictive performance. **Results**: A total of 1274 patients met the inclusion criteria. Compared to non-PPMI patients with early cardiac damage stage, those with PPMI and advanced stage had higher all-cause mortality (HR: 2.98; 95% CI: 1.60–5.56; *p* value < 0.001) and more cardiac deaths (HR: 3.85; 95% CI: 1.36–11.10; *p* = 0.012). Multivariable analysis confirmed PPMI and cardiac damage stage as independent prognostic factors. Notably, the predictive model incorporating both variables achieved the best performance (AUC = 0.703) with significant NRI and favorable DCA results, suggesting incremental value for identifying high-risk patients and acceptable clinical utility. **Conclusions**: The current study is among the first to explore whether post-procedural cardiac damage may identify a subgroup in whom PPMI carries a particularly adverse long-term prognosis, highlighting the need for integrated risk assessment for post-TAVR lifelong management.

## 1. Introduction

Transcatheter aortic valve replacement (TAVR) has become a first-line treatment for severe symptomatic aortic stenosis (AS) at all levels of risk [1,2,3]. As indications expand to younger, lower-risk patients, the focus has shifted toward lifetime management strategies and sustained functional improvement [4]. Nevertheless, post-procedural conduction disturbances (CDs) remain common and often necessitate permanent pacemaker implantation (PPMI) [5], which predicts adverse outcomes [6,7,8,9,10,11]. The resultant electromechanical dyssynchrony progressively impairs ventricular function and promotes adverse remodeling [12,13,14]. Moreover, this deleterious effect is amplified in patients with pre-existing cardiac dysfunction. Indeed, pooled analysis of NEOPRO registries revealed that patients with concomitant left ventricular dysfunction (LVEF < 40%) and PPMI experienced higher 1-year mortality relative to those with PPMI alone [15], underscoring a critical interaction between pacing and cardiac function.

However, LVEF alone is insufficient to capture the full spectrum of cardiac dysfunction in TAVR patients, as chronic pressure overload from AS causes heterogeneous cardiac damage that may persist after valve replacement. The five-stage cardiac damage classification proposed by Généreux et al. provides a structured framework to quantify pre-procedural cardiac damage burden, with higher stages showing a stepwise mortality increase [16,17,18,19]. Importantly, because TAVR rapidly relieves pressure overload and alters intracardiac hemodynamics [20,21], recent evidence shows that the post-procedural cardiac stage has even stronger prognostic implications in patients with severe AS, up to five years after TAVR [17].

Given the heterogeneity in cardiac function and its potential to modify PPMI-associated risks, the interplay between these factors for accurate risk stratification remains unclear. The current study sought to ascertain the combined risk of PPMI and post-TAVR cardiac damage on long-term outcomes and to develop a predictive model for long-term prognosis, offering insights for clinical management.

## 2. Materials and Methods

### 2.1. Study Population

This was a retrospective, single-center study. Patients who underwent TAVR at the West China Hospital of Sichuan University from July 2013 to June 2023 were enrolled consecutively. The multidisciplinary heart team assessed all patients for TAVR eligibility, with valve type selection based on device availability and clinical status at the time of intervention. Key exclusion criteria included (1) those with pre-procedural PPMI; (2) those with missing pre- or post-procedural electrocardiographic data; and (3) those with missing pre- or post-procedural echocardiographic data required for the assessment of cardiac stage. This study was approved by the Biomedical Ethics Review Committee of West China Hospital, Sichuan University, and conducted in adherence to the Declaration of Helsinki principles.

### 2.2. Patient Data Collection

Baseline and periprocedural characteristics were collected from electronic patient records, including demographics, laboratory parameters, and relevant comorbidities. Pre- and post-procedural electrocardiographic and echocardiographic data were validated in all patients. The indication for PPMI within 30 days post-TAVR, as well as selection of pacemaker types and pacing mode, was determined according to ACC/AHA/HRS guidelines [22]. Patients were classified into 5 cardiac damage stages according to the proposed system [23]: Stage 0, no other cardiac damage seen; Stage 1, LV damage (LVEF < 60%, LV mass index > 95 g/m^2^ for women, >115 g/m^2^ for men, or E/e′ ≥ 14); Stage 2, left atrium or mitral valve damage (presence of enlarged left atrium or atrial fibrillation, or mitral regurgitation ≥ moderate); Stage 3, pulmonary artery vasculature or tricuspid valve damage or dysfunction (pulmonary artery systolic pressure (PASP) ≥ 60 mmHg or tricuspid regurgitation ≥ moderate); and Stage 4, RV damage, as defined by the presence of moderate to severe RV dysfunction (tricuspid annular plane systolic excursion (TAPSE) < 16 mm). All echocardiograms were reviewed for accuracy by experienced echocardiologists. Patients who met the criteria for multiple stages were assigned to the highest (most severe) stage. Subsequently, they were reclassified into the early stage (Stages 0–2) and advanced stage (Stages 3–4) according to previously proposed principles [24]. All echocardiograms were analyzed in a blinded core-lab fashion. The cardiac stage classification was performed locally by our research team based on the core-lab echocardiographic data, at discharge (*n* = 48 [3.8%]) or at 30 days (*n* = 1226 [96.2%]). Stage classification was initially performed by two independent cardiologists (R.T. and X.Y.) and then reviewed by a senior cardiologist (Y.Y.) to ensure consistency. The post-TAVR cardiac stage was assessed by echocardiography at 30 days or hospital discharge.

### 2.3. Outcomes

The primary endpoint of this study was all-cause mortality. And the secondary endpoint includes cardiovascular mortality at 3 years. All clinical endpoints were adjudicated according to current Valve Academic Research Consortium-3 criteria [25]. Regular clinical follow-up was scheduled for 30 days, 1 year, 2 years, and 3 years after TAVR. Data were obtained by standardized interviews, documentation from specialized physicians, and hospital discharge summaries.

### 2.4. Statistical Analysis

For two-group comparisons, continuous variables are presented as the median (IQR) and were compared using the Mann–Whitney U test or Student’s *t*-test, as appropriate. Categorical variables are presented as absolute values along with percentages and were compared using the Pearson χ^2^ test or Fisher exact test when appropriate. For comparisons involving more than two groups, continuous variables were compared using the Kruskal–Wallis test. For within-group comparisons across different time points, overall differences were evaluated using the Friedman test. Post hoc pairwise comparisons against the baseline were performed using the paired Wilcoxon signed-rank test, and the resulting *p* values were adjusted for multiple testing using the Bonferroni method. A corrected two-sided *p* value < 0.025 was considered statistically significant for these post hoc analyses. Time-to-event curves were constructed using the Kaplan–Meier method from the date of the procedure onward. Patients were classified into 4 groups for survival analysis: (1) Group A: patients with only early stage of cardiac damage but without PPMI within 30 days after TAVR; (2) Group B: patients with early stage of cardiac damage but with PPMI within 30 days after TAVR; (3) Group C: patients with advanced stage of cardiac damage but without PPMI within 30 days; (4) Group D: patients with both advanced stages and PPMI at 30-day post-TAVR. Multivariable Cox proportional hazards models were used to calculate HRs and 95% CIs for the clinical outcomes, with the proportional hazards assumption verified. And the multivariable analysis was performed with adjustment for age, sex, body mass index (BMI), Society of Thoracic Surgeons Predicted Risk of Mortality score (STS score), hypertension, diabetes mellitus, chronic kidney disease (CKD), chronic obstructive pulmonary disease (COPD), coronary artery disease (CAD), and peripheral artery disease (PAD). To evaluate the incremental predictive value of cardiac damage and PPMI rate on long-term outcomes after transcatheter aortic valve replacement (TAVR), we constructed time-dependent receiver operating characteristic (ROC) curves and calculated the corresponding C-index. Pairwise model comparisons were conducted using the DeLong test. Also, the prediction model’s sensitivity, specificity, and clinical applicability were evaluated using time-dependent AUC, classical net reclassification improvement (NRI), integrated discrimination improvement (IDI), calibration tests with bootstrap internal validation, and decision curve analysis (DCA). A two-sided *p* value < 0.05 yielded statistical significance. All statistical analyses were performed with the use of SPSS statistical package version 27.0 (IBM SPSS Statistics for Windows, Armonk, NY, USA) and R software version 4.4.1 (R Project for Statistical Computing).

## 3. Results

### 3.1. Study Population and Baseline Characteristics

**Figure 1:**
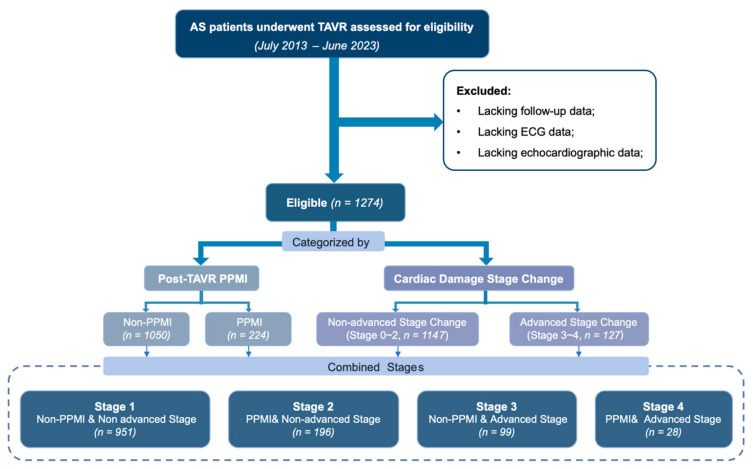
**Study Flow Chart. Abbreviations:** AS = aortic stenosis; PPMI = permanent pacemaker implantation; and TAVR = transcatheter aortic valve replacement.

**Figure 2:**
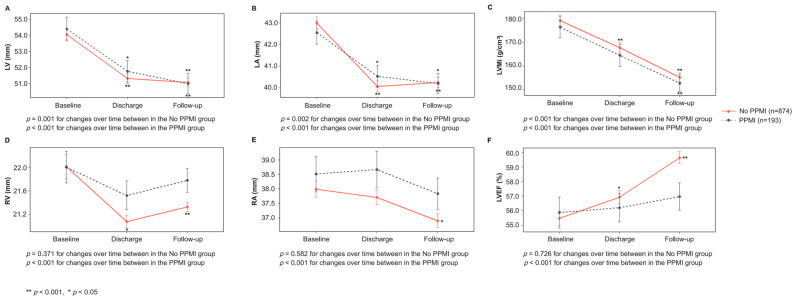
**Cardiac structural remodeling in patients with and without a permanent pacemaker.** Changes in chamber dimensions over time are shown for (**A**) LV, (**B**) LA, (**C**) LVMi, (**D**) RV, (**E**) RA, and (**F**) LVEF at baseline, discharge, and 30-day follow-up in the PPMI and non-PPMI groups. **Abbreviations:** LV = left ventricle; LA = left atrium; LVMi = left ventricular mass index; RV = right ventricle; RA = right atrium; LVEF = left ventricular ejection fraction; and PPMI = permanent pacemaker implantation.

A total of 1274 patients were included in this study (Figure 1). The mean age was 73.0 years [IQR, 69.0–79.0], and 58.0% of them were males. Of these, 224 patients (17.6%) required PPMI within 30 days post-TAVR, with the majority (86.2%) receiving DDD-mode devices. Most TAVR recipients were at an early stage in terms of cardiac damage. In brief, patients with PPMI were older (73.0 [IQR, 68.0–78.0] years of age vs. 74.0 [IQR, 69.0–79.0] years of age; *p* = 0.012) and had higher rates of CKD (54 [5.1%] vs. 21 [9.4%]; *p* = 0.015, see Table S1). Baseline characteristics for patients with different cardiac damage stages are presented in Table S2. Baseline echocardiographic parameters were also observed differently among the cohort. At 30 days post-TAVR, remodeling of cardiac structures was observed in both patients with and without PPMI (Figure 2). However, reduction in right-sided heart dimensions was exclusive to the non-PPMI group compared with the baseline (RV, *p* value < 0.001; RA, *p* = 0.007). A concurrent increase in left ventricular ejection fraction was also observed in this group (*p* value < 0.001).

### 3.2. Risk Factors and Patient Grouping for Long-Term Outcomes

Cox regression analysis was used to analyze the long-term prognosis post-TAVR. Univariate and multivariable survival analyses for the total study population are presented in Table 1. Age, STS score, hypertension, COPD, CKD, CAD, cardiac damage, and PPMI within 30 days were associated with higher 3-year mortality rates in univariate analyses. Following the multivariable adjustment, cardiovascular damage stage and PPMI within 30 days post-TAVR were still found to be independent risk factors for all-cause mortality.

Based on the status of cardiac damage and PPMI, four groups were identified: 951 non-PPMI patients with early stage of cardiac damage (Group A), 196 PPMI patients with early stage of cardiac damage (Group B), 99 non-PPMI patients with advanced stage of cardiac damage (Group C), and 28 PPMI patients with advanced stage of cardiac damage (Group D). The baseline characteristics stratified by groups generally aligned with the levels of cardiac damage, except for gender (Table 2). The combination of PPMI and advanced cardiac damage was associated with older age and higher New York Heart Association (NYHA) class (*p* value < 0.001). Compared with the other groups, patients in Group D had more comorbidities, including dialysis (e.g., Group A, 7 [0.7%] vs. Group D, 4 [14.3%]), atrial fibrillation (e.g., Group A, 113 [11.9%] vs. Group D, 10 [35.7%]), hypertension (e.g., Group A, 436 [45.8%] vs. Group D, 14 [50.0%]), and had a higher STS score (e.g., Group A, 3.2% [IQR, 2.2–6.0%] vs. Group D, 4.8% [IQR, 2.5–10.3%]).

### 3.3. Post-TAVR Clinical Outcomes and Survival Analysis at 3 Years

During a median follow-up of 3 years after TAVR, a total of 274 (21.5%) deaths occurred in the entire cohort. Patients with advanced cardiac stage or PPMI tended to have higher rates of all-cause death (cardiac stage, *p* = 0.001; PPMI, *p* = 0.004), with a stepwise increase in cardiovascular mortality at 3 years according to progressive stages of cardiac damage exclusively (*p* = 0.006, see Figure S1). At 3 years, all-cause death occurred in 19.4% (185/951) of patients in Group A, 24.4% (48/196) in Group B, 30.3% (30/99) in Group C, and 39.2% (11/28) in Group D. Cardiovascular death occurred in 6.9% (66/951) of Group A, 6.6% (13/196) of Group B, 11.1% (11/99) of Group C, and 14.2% (4/28) of Group D (both *p* value < 0.001, see Table S3).

**Figure 3:**
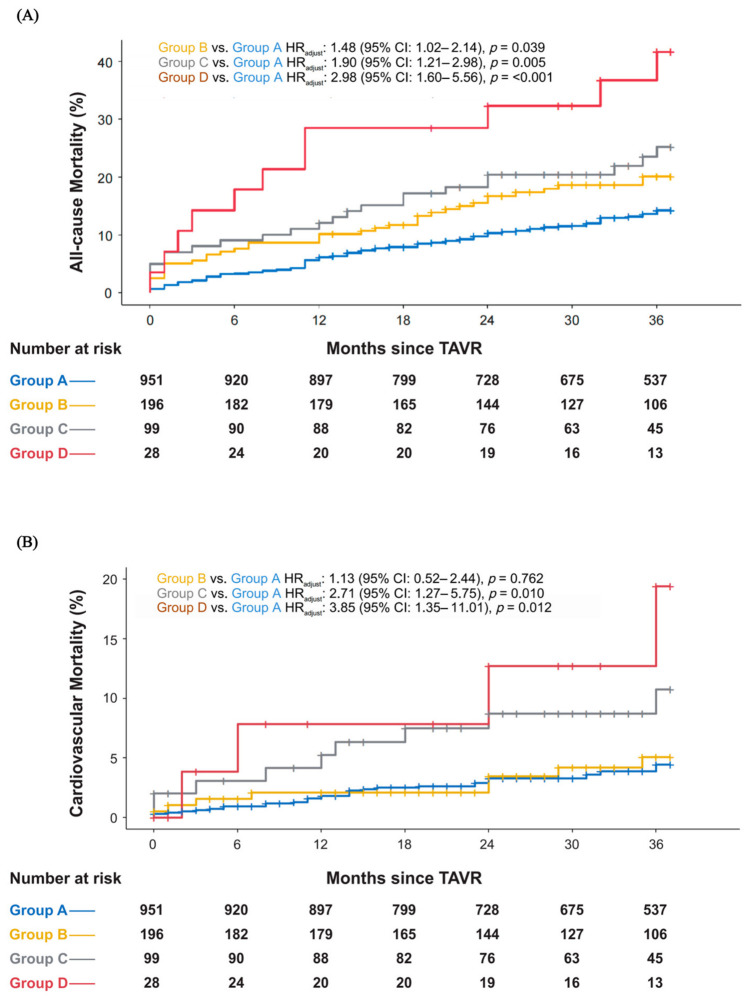
**Association of PPMI and cardiac damage with follow-up outcomes.** All-cause deaths (**A**) and cardiovascular deaths (**B**) are presented as stratified by the four groups based on PPMI and cardiac damage status. The abbreviations are the same as those in Figure 1 and Figure 2.

Compared with patients in Group A, those in Groups B, C, and D had higher all-cause mortality after multivariable adjustments (Group B: HR, 1.48; 95% CI: 1.02–2.14; *p* = 0.039; Group C: HR, 1.90; 95% CI: 1.14–2.87; *p* = 0.005; and Group D: HR, 2.98; 95% CI: 1.60–5.56; *p* value < 0.001, see Figure 3A). Regarding cardiovascular mortality at 3 years, patients in Groups C and D had a higher incidence of mortality compared with patients in Group A (HR: 2.71; 95% CI: 1.27–5.75; *p* = 0.010 and HR: 3.85; 95% CI: 1.35–11.01; *p* = 0.012, see Figure 3B). To assess whether the prognostic effect of the combined grouping was modified by temporal changes in TAVR devices and practice, we performed a sensitivity analysis stratified by implantation era. We fitted the multivariable Cox model with interaction terms between the four combined groups and device generation. All interaction terms were non-significant (all *p* values for interaction > 0.05), indicating that the association between the combined grouping and 3-year mortality remained consistent across the two eras.

### 3.4. The Incremental Predictive Value of PPM-Cardiac Damage Combined Stage for Long-Term Prognosis

**Figure 4:**
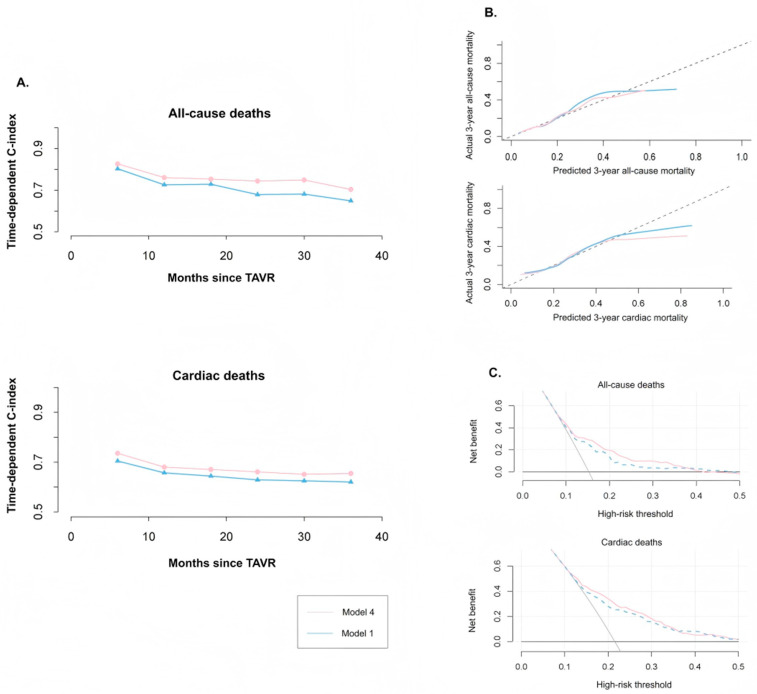
**Construction and validation of prognostic models.** (**A**) Time-dependent ROC curve of Model 4 and Model 1 (**upper**: all-cause mortality; **lower**: cardiac mortality). Circles and triangles denote Model 4 and Model 1, respectively. (**B**) Calibration curve: The calibration plot indicated acceptable agreement between observed and predicted risks overall, although a slight overestimation was observed for patients with intermediate-to-high predicted risks. The diagonal dashed line indicates perfect agreement between predicted and observed probabilities. (**C**) Clinical practicability DCA curve. The pink solid and blue dashed curves represent Model 4 and Model 1, respectively. **Abbreviations:** Time-ROC = time-dependent receiver operating characteristic curve; and DCA = decision curve analysis curve. The other abbreviations are the same as those in Figure 1 and Figure 2.

Next, related prognostic models containing different risk factors were established. The base model (Model 1) included only established clinical factors. To evaluate additive prognostic contributions of PPMI and cardiac damage, three expanded models were built: Model 2 incorporated PPMI into Model 1; Model 3 incorporated cardiac damage into Model 1; and Model 4 incorporated both cardiac damage and PPMI into the baseline model (Table 3). The time-dependent ROC curve and calibration (bootstrap internal validation) were used to verify the accuracy of the model (Figure 4A,B). The C-index of Model 4 that was used to predict all-cause mortality was calculated to be 0.703 (95% CI 0.665–0.753), and 0.687 (95% CI 0.645–0.729) for cardiac mortality (Figure 4A). Time-dependent ROC curve analyses at 3 years showed that the AUC value of Model 4 was the highest and significantly better than that of Model 1, for all-cause death (AUC: 0.703 vs. 0.670, *p* < 0.001) and cardiac death (AUC: 0.667 vs. 0.640, *p* = 0.006), suggesting its potential value for 3 y outcome prediction (Table 3). With regard to the comparison of the NRI of 3-year survival between Model 4 and Model 1, the NRI for all-cause mortality was 0.051 (95% CI 0.004–0.101, *p* = 0.035) for 3-year deaths, and 0.009 (95% CI −0.008–0.025, *p* = 0.285) for 3-year cardiac deaths, which means approximately 5 patients per 100 individuals for the combined endpoint. In addition, the IDI of Model 4 was 0.015 (95% CI 0.008–0.024, *p* < 0.001) for all-cause death and 0.004 (95% CI 0.001–0.007, *p* = 0.002) for cardiac death. The 3-year DCA curve also indicated that Model 4 provided a higher net benefit across a broad range of threshold probabilities (Figure 4C), supporting its potential clinical usefulness despite the modest NRI/IDI values.

## 4. Discussion

This study is among the first to investigate the combined impact of PPMI and post-TAVR cardiac damage on long-term outcomes in severe aortic stenosis patients. Our major findings are as follows: (1) Both PPMI and cardiac damage stage were identified as independent risk factors for long-term prognosis. (2) At 30-day follow-up, patients with or without PPMI demonstrated significant cardiac remodeling from echocardiographic data, yet improved right heart dimensions were exclusively observed in patients without PPMI. (3) A stepwise increase in 3-year all-cause mortality was observed in patients from Group A (no PPMI + early damage) to Group D (PPMI + advanced damage), demonstrating that coexisting PPMI and severe cardiac damage indicated high risk. (4) The predictive model integrating both factors outperformed traditional models in discrimination, reclassification, and clinical net benefit. Our findings highlight the importance of novel risk factors that contribute considerably to the residual risk after TAVR. Incorporating these factors into routine clinical assessments may improve the identification of high-risk individuals and facilitate the development of targeted prevention strategies.

Relief of pressure overload by TAVR leads to immediate changes in intracardiac hemodynamics, potentially altering the assessment of cardiac disease stage in the post-TAVR period. Therefore, the pre-procedural stage may not fully reflect the post-interventional prognosis. In fact, any residual or new-onset cardiac damage after TAVR often holds greater clinical relevance for patient prognosis than the pre-procedural stage alone, a consideration underscored by the potential for reverse remodeling in the long term. This concept is supported by Avvedimento et al., who demonstrated that cardiac damage stage may change early after TAVI, with a lower prevalence of advanced cardiac damage at 30 days, mainly driven by improvements in left ventricular diastolic parameters and right ventricular function [26]. Our analysis was in accordance with previous findings. Echocardiographic evaluation at 30-day follow-up revealed significant cardiac remodeling in most patients, involving parameters of both ventricles, albeit with differences between subgroups, indicating potential long-term outcome differences.

New PPMI after TAVR has been associated with several complications, including tricuspid regurgitation, device infection, lead failure, pocket erosion, pocket hematoma, and right ventricular perforation [27], which may be related to worse clinical outcomes in selected patients. However, conflicting evidence exists concerning the long-term clinical impact of PPMI following TAVR. A recent meta-analysis including 42,927 patients (21 studies) evaluated the impact of PPMI and illustrated that periprocedural pacemaker implantation after TAVR was associated with a higher risk of all-cause death and heart failure hospitalization but was not associated with increased cardiac death [11]. Another comprehensive systematic search assessing the short- and long-term clinical outcomes in patients undergoing PPMI post-TAVR aligned with this result. On the contrary, the SwissTAVI Registry, encompassing 13,360 patients, demonstrated that patients with PPMI exhibited an LVEF decline ≥10% and worse NYHA class function, along with higher all-cause and cardiovascular mortality rates at 5- and 10-year follow-up [10]. Experience from the PARTNER 2 S3 registries showed no association with adverse clinical outcomes in patients with PPMI through 5 years [6]. And our findings are in accordance with the results of the meta-analysis. Such discrepancies may be attributed to multiple factors, including the differences in valve types and characteristics of the patient populations in different studies, as well as confounding effects [28].

Our study showed that the association between new PPMI and 3-year all-cause mortality appeared more pronounced in patients with more advanced cardiac damage. Patients with more advanced damage had a higher mortality risk than those with less damage despite also receiving PPMI, highlighting the critical role of post-TAVR cardiac function. Of note, because PPMI status and post-TAVR cardiac damage stage were assessed at the same time point (30 days after TAVR), we could not determine whether PPMI causally modified the staging or whether the observed interaction simply reflects a higher baseline burden of cardiac dysfunction. However, previous studies have suggested that chronic right ventricular pacing may be associated with impaired systolic function through pacing-induced electromechanical dyssynchrony, altered ventricular contraction patterns, and paradoxical interventricular septal motion [29,30,31]. It also causes dyssynchrony of the papillary muscles, with additional functional impairment, such as increased LV volume and mitral regurgitation [32,33,34,35]. These pacing-related mechanisms may partly underlie the observed association between PPMI and poorer functional status or higher risk of heart failure hospitalization after TAVR, as reported in previous studies [36,37,38]. This is particularly prominent in patients with pre-existing LV impairment. Patients with pre-existing cardiac dysfunction are more sensitive to these adverse effects, with more impairment in the left atrial emptying, which harms ventricular filling despite maintaining the atrial–ventricular sequence [30,39,40]. Previous studies have demonstrated that new PPMI was associated with 1-year outcomes in patients with LV dysfunction, showing a higher risk of major adverse cardiac events in subgroups with baseline LVEF < 40% [15]. Furthermore, right ventricular dysfunction, as a marker of advanced cardiac damage, may increase vulnerability to pacing-related dyssynchrony and impaired remodeling, which could partly account for the high-risk profile observed in patients with both new PPMI and advanced cardiac damage. This is supported by evidence from cardiac resynchronization therapy (CRT) studies: in patients with very severe cardiac dysfunction, those with RV dysfunction showed significantly less improvement at 18 months after CRT compared to those without RV dysfunction, and such patients may be considered “too sick” to achieve optimal LV reverse remodeling benefits [41]. With the expansion of TAVR into younger and lower-risk populations, careful assessment of cardiac function and post-procedural risk may help identify patients who require closer follow-up after new PPMI.

Despite the strengths of this nationally representative cohort, several limitations should be acknowledged. First, due to the retrospective and single-center nature of this study, it was exposed to a risk of selection bias, and its findings may be of limited generalizability to other populations. Multicenter studies are needed to further validate these findings and explore their applicability across diverse populations and healthcare settings. Second, the subgroup with both advanced cardiac damage and PPMI was relatively small. To mitigate the associated risks of statistical instability and model overfitting, we performed internal validation using a bootstrap approach for calibration. Nevertheless, the sample size may still limit the robustness of multivariable analyses and hazard estimates. Third, pacing burden and medical therapy at follow-up were not present for the totality of patients. Fourth, the predominance of self-expanding valves in our cohort limits the generalizability of our findings to populations with a more balanced distribution of valve types. However, valve type did not differ significantly between patients with and without PPMI, suggesting that this imbalance is unlikely to have substantially biased the main comparisons. Finally, future studies with serial echocardiographic and device follow-up are needed to clarify whether PPMI contributes to cardiac damage progression after TAVR.

## 5. Conclusions

In patients undergoing TAVR, the coexistence of post-procedural advanced cardiac damage and permanent pacemaker implantation identifies subgroups at markedly elevated long-term mortality risk, highlighting the importance of comprehensive evaluation and tailored management strategies for prognostic risk stratification.

## Figures and Tables

**Table 1:** Univariable and multivariable Cox proportional hazards analysis on long-term survival for TAVR recipients.

Variables	Univariable		Multivariable	
	HR (95% CI)	*p*	HR (95% CI)	*p*
**Age**	1.03 (1.01–1.05)	0.003	1.02 (1.00–1.04)	0.066
**Gender**	0.80 (0.60–1.07)	0.134		
**BMI**	0.95 (0.92–0.99)	0.007	0.95 (0.91–0.99)	0.017
**STS score**	1.07 (1.05–1.09)	<0.001	1.03 (1.00–1.06)	0.026
**NYHA class**	1.12 (0.93–1.36)	0.233		
**Hypertension**	1.26 (0.98–1.62)	0.071	1.15 (0.85–1.56)	0.358
**Diabetes**	1.13 (0.84–1.53)	0.417		
**COPD**	1.52 (1.17–1.97)	0.002	0.85 (0.75–1.19)	0.341
**Atrial fibrillation**	0.87 (0.60–1.26)	0.446		
**CKD**	2.28 (1.52–3.42)	<0.001	1.48 (0.91–2.42)	0.059
**CAD**	1.31 (0.99–1.74)	0.055	1.08 (0.78–1.76)	0.626
**Albumin**	1.04 (0.96–1.12)	0.324		
**eGFR**	0.99(0.99–1.00)	0.218		
**PPMI**	1.44 (1.07–1.95)	0.002	1.31 (1.09–1.57)	0.015
**PAD**	1.221(0.85–1.75)	0.275		
**Cardiac damage stage**	1.30 (1.11–1.51)	0.001	1.55 (1.11–2.16)	0.008

**Abbreviations:** CKD: chronic kidney disease; PAD: peripheral artery disease; COPD: chronic obstructive pulmonary disease; CAD: coronary artery disease; STS score: Society of Thoracic Surgeons Predicted Risk of Mortality score; NYHA class: New York Heart Association class; HR: hazard ratio; and 95% CI: 95% confidence interval.

**Table 2:** Baseline characteristics of participants by cardiac damage change and PPMI combined groups.

Characteristics	Cardiac Damage and PPMI Combined Groups				*p* Value
	Early Stage		Advanced Stage		
	Without PPMI (Group A) N = 951	With PPMI (Group B) N = 196	Without PPMI (Group C) N = 99	With PPMI (Group D) N = 28	
**Age (y)**	73.0 (68.0, 77.0)	73.5 (68.0, 78.5)	74.0 (68.0, 78.0)	75.0 (71.5, 80.5)	0.019
**Gender, n (%)**					0.048
**Male**	551 (57.9%)	126 (64.3%)	47 (47.5%)	15 (53.6%)	
**Female**	400 (42.1%)	70 (36.7%)	52 (53.5%)	13 (46.4%)	
**BMI (kg/m^2^)**	22.9 (20.4, 25.2)	23.0 (21.1, 25.0)	21.6 (19.1, 24.8)	21.5 (20.2, 24.1)	0.093
**Hypertension, n (%)**	436 (45.8%)	86 (43.9%)	26 (26.3%)	14 (50.0%)	0.002
**Diabetes, n (%)**	194 (20.4%)	47 (24.0%)	23 (23.2%)	9 (32.1%)	0.327
**NYHA class, n (%)**					<0.001
**1**	10 (1.0%)	4 (2.0%)	1 (1.0%)	0 (0.0%)	
**2**	275 (28.9%)	59 (30.1%)	10 (10.1%)	4 (14.3%)	
**3**	555 (58.4%)	114 (58.2%)	55 (55.6%)	18 (64.3%)	
**4**	111 (11.7%)	19 (9.7%)	33 (33.3%)	6 (21.4%)	
**STS score (%)**	3.2 (2.2, 6.0)	3.1 (2.0, 5.2)	4.3 (2.6, 7.8)	4.8 (2.5, 10.3)	0.006
**Creatinine clearance (mL/min)**	53.6 (41.2, 67.6)	48.7 (38.0, 63.3)	51.4 (38.1, 63.8)	39.4 (23.7, 52.8)	<0.001
**eGFR (mL/min/1.73 m^2^)**	69.2 (54.6, 85.7)	65.1 (50.7, 82.0)	68.0 (51.6, 85.9)	51.8 (32.3, 70.7)	0.001
**Chronic obstructive pulmonary disease, n (%)**	234 (24.6%)	58 (29.6%)	24 (24.2%)	6 (21.4%)	0.487
**Cardiovascular disease, n (%)**	154 (16.2%)	24 (12.2%)	14 (14.1%)	5 (17.9%)	0.527
**Chronic kidney disease, n (%)**	44 (4.6%)	16 (8.2%)	10 (10.1%)	5 (17.8%)	0.003
**Peripheral artery disease, n (%)**	155 (16%)	31 (16%)	13 (13%)	5 (17.9%)	0.860
**Dialysis, n (%)**	7 (0.7%)	1 (0.5%)	1 (1.0%)	4 (14.3%)	<0.001
**Coronary artery disease, n (%)**	227 (23.9%)	47 (24.0%)	27 (27.3%)	9 (32.1%)	0.679
**Prior myocardial infarction, n (%)**	18 (1.9%)	3 (1.5%)	1 (1.0%)	1 (3.6%)	0.713
**Prior atrial fibrillation, n (%)**	113 (11.9%)	26 (13.3%)	35 (35.4%)	10 (35.7%)	<0.001
**Cancer, n (%)**	26 (2.7%)	7 (3.6%)	2 (2.0%)	0 (0.0%)	0.836
**Pre-procedural LVEF (%)**	55.6 (14.7)	57.0 (14.3)	54.5 (15.2)	50.0 (16.0)	0.085
**Valve type, n (%)**					
**Self-expanding valve**	765 (91.4%)	160 (90.4%)	77 (88.5%)	23 (95.8%)	
**Balloon-expandable valve**	62 (7.4%)	13 (7.3%)	8 (9.2%)	0 (0.0%)	
**Mechanically expandable valve**	10 (1.2%)	4 (2.3%)	2 (2.3%)	1 (4.2%)	
**V max (m/s)**	4.7 (4.2, 5.2)	4.7 (4.3, 5.3)	4.7 (4.0, 5.2)	4.7 (3.8, 5.5)	0.287
**MPG (mm Hg)**	53.0 (42.0, 72.0)	56.0 (46.0, 72.0)	53.0 (40.0, 66.0)	49.0 (32.0, 71.0)	0.126
**LV (mm)**	51.0 (46.0, 58.0)	50.0 (45.0, 59.0)	50.0 (45.0, 59.0)	52.0 (47.0, 61.0)	0.858
**RV (mm)**	21.0 (20.0, 23.0)	21.0 (20.0, 23.0)	22.0 (20.0, 24.0)	22.0 (20.0, 24.0)	0.344
**LA (mm)**	42.0 (38.0, 47.0)	41.0 (37.0, 47.0)	45.0 (40.0, 50.0)	45.0 (38.0, 50.0)	0.001
**RA (mm)**	36.0 (33.0, 40.0)	36.0 (33.0, 40.0)	42.0 (35.0, 47.0)	41.0 (33.0, 47.0)	<0.001
**IVS (mm)**	13.0 (12.0, 15.0)	13.0 (12.0, 14.0)	13.0 (12.0, 15.0)	13.0 (12.0, 15.0)	0.326
**LVPW (mm)**	12.0 (10.0, 13.0)	12.0 (10.0, 12.0)	12.0 (10.0, 13.0)	11.0 (10.0, 12.0)	0.400

**Abbreviations:** NYHA class: New York Heart Association class; STS score: Society of Thoracic Surgeons Predicted Risk of Mortality score; eGFR: estimated glomerular filtration rate; BMI: body mass index; LVEF: left ventricular ejection fraction; LV: left ventricle; RV: right ventricle; LA: left atrium; RA: right atrium; IVS: interventricular septum; LVPW: left ventricular posterior wall; MPG: mean pressure gradient; PPMI: permanent pacemaker implantation.

**Table 3:** Comparison of model performance.

	Different Models					
	AUC	*p* Value (vs. Model 1)	NRI	*p* Value (vs. Model 1)	IDI	*p* Value (vs. Model 1)
**3 y Death**						
**Model 1**	0.670 (0.638–0.703)	-	-	-	-	-
**Model 2**	0.688 (0.651–0.725)	0.012 *	0.032 (−0.006–0.071)	0.101	0.004 (0.000–0.009)	0.059
**Model 3**	0.695 (0.662–0.730)	0.005 *	0.028 (−0.014–0.070)	0.187	0.011 (0.004–0.017)	<0.001 *
**Model 4**	0.703 (0.670–0.741)	<0.001 *	0.051 (0.004–0.101)	0.035 *	0.015 (0.008–0.024)	<0.001 *
**3 y Cardiac Death**						
**Model 1**	0.640 (0.602–0.681)	-				
**Model 2**	0.645 (0.606–0.684)	0.063	0.004 (−0.004–0.011)	0.316	0.000 (0.000–0.001)	0.620
**Model 3**	0.665 (0.624–0.703)	0.006 *	0.010 (0.006–0.026)	0.231	0.004 (0.002–0.007)	0.002 *
**Model 4**	0.667 (0.629–0.705)	0.006 *	0.009 (−0.008–0.025) *	0.285	0.004 (0.001–0.007)	0.002 *

The base model (Model 1) included only established clinical factors, such as age, sex, body mass index (BMI), Society of Thoracic Surgeons Predicted Risk of Mortality score (STS score), hypertension, diabetes mellitus, chronic kidney disease (CKD), chronic obstructive pulmonary disease (COPD), coronary artery disease (CAD), peripheral artery disease (PAD), NYHA class, and eGFR. To evaluate additive prognostic contributions of PPMI and cardiac damage, three expanded models were built: Model 2 incorporated PPMI into Model 1; Model 3 incorporated cardiac damage into Model 1; and Model 4 incorporated both cardiac damage and PPMI into the baseline model. * indicates *p* value < 0.05. Abbreviations: IDI: incremental discriminative improvement; NRI: net reclassification improvement; and AUC: area under the curve. Other abbreviations are the same as those in Table 1 and Table 2.

## Data Availability

The data presented in this study are available upon request from the corresponding authors.

## Supplementary Materials

The following supporting information can be downloaded at: https://www.mdpi.com/article/10.3390/biomedicines14071569/s1, Table S1: Baseline Characteristics of Participants stratified by Postprocedural PPMI; Table S2: Baseline Characteristics of Participants Stratified by Cardiac Damage; Table S3: Clinical Outcomes According to Cardiac Damage combined with PPMI Classification at 3-y follow-up; Figure S1: Kaplan-Meier curves of 3y outcomes for cardiac damage and PPM separately.

## Abbreviations

The following abbreviations are used in this manuscript:

AS	Aortic Stenosis
PPMI	Permanent Pacemaker Implantation
TAVR	Transcatheter Aortic Valve Replacement
NYHA	New York Heart Association
STS	Society of Thoracic Surgeons
eGFR	Estimated Glomerular Filtration Rate
LVEF	Left Ventricular Ejection Fraction
CKD	Chronic Kidney Disease
PAD	Peripheral Artery Disease
COPD	Chronic Obstructive Pulmonary Disease
CAD	Coronary Artery Disease
AUC	Area Under the Curve
LV	Left Ventricle
RV	Right Ventricle
LA	Left Atrium
RA	Right Atrium
IVS	Interventricular Septum
LVPW	Left Ventricular Posterior Wall
IDI	Incremental Discriminative Improvement
NRI	Net Reclassification Improvement
