# Supplementary material for: Integrated Impact of Post-TAVR Cardiac Damage and Pacemaker Implantation on Long-Term Outcomes
Source: Biomedicines. 2026 Jul 13;14(7):1569. doi: 10.3390/biomedicines14071569 (PMC13406656; doi:10.3390/biomedicines14071569)
Supplement: Supplementary file 1 [file biomedicines-14-01569-s001.zip › Sup Table S3-clinical outcome HR.pdf]

Table S3 Clinical Outcomes According to Cardiac Damage combined with PPMI Classification at 3-y follow-up

Clinical Outcomes According to Group Classification						
	Group A (N=951)	Group B (N=196)	Group C (N=99)	Group D (N=28)	Linear Trend HR (95% CI)	*P* Value
At 3-year						
All-cause mortality	185 (19.4%)	48 (24.4%)	30 (30.3%)	11 (39.2%)	1.46 (1.25-1.71)	<0.001
Cardiovascular mortality	66 (6.9%)	13 (6.6%)	11 (11.1%)	4 (14.2%)	1.60 (1.21-2.10)	<0.001

Abbreviations: HR: Hazard Ratio; CI: Confidence Interval. The rest are in accordance with Table S1&2.
