# Supplementary material for: Integrated Impact of Post-TAVR Cardiac Damage and Pacemaker Implantation on Long-Term Outcomes
Source: Biomedicines. 2026 Jul 13;14(7):1569. doi: 10.3390/biomedicines14071569 (PMC13406656; doi:10.3390/biomedicines14071569)
Supplement: Supplementary file 1 [file biomedicines-14-01569-s001.zip › Sup Table S2.pdf]

Table S2 Baseline Characteristics of Participants Stratified by Cardiac Damage

Characteristics	Cardiac damage Status: Early Stage (0~2) N = 1,147	Advanced Stage (3~4) N = 127	*P* value
**Age (y)**	73 (68, 78)	74 (68, 78)	0.072
**Gender, n (%)**			0.027
Male	677 (59%)	62 (49%)	
Female	470 (41%)	65 (51%)	
**BMI (kg/m²)**	22.9 (20.5, 25.2)	21.5 (19.4, 24.7)	0.016
**Hypertension, n (%)**	522 (46%)	40 (31%)	0.003
**Diabetes, n (%)**	241 (21%)	32 (25%)	0.275
**NYHA Class, n (%)**			<0.001
1	14 (1.2%)	1 (0.8%)	
2	334 (29%)	14 (11%)	
3	669 (58%)	73 (57%)	
4	130 (11%)	39 (31%)	
**STS Score (%)**	3.2 (2.2, 6.0)	4.5 (2.6, 8.3)	<0.001
**Creatinine Clearance (mL/min)**	53 (41, 67)	49 (33, 62)	0.005
**eGFR, (mL/min/1.73 m²)**	69 (54, 85)	65 (43, 81)	0.030
**Chronic Obstructive Pulmonary Disease, n (%)**	292 (25%)	30 (24%)	0.652
**Cardiovascular Disease, n (%)**	178 (16%)	19 (15%)	0.869
**Chronic Kidney Disease, n (%)**	60 (5.2%)	15 (12%)	0.003
**Peripheral Vascular Disease, n (%)**	186 (16%)	18 (14%)	0.551
**Dialysis, n (%)**	8 (0.7%)	5 (3.9%)	0.006
**Coronary Artery Disease, n (%)**	274 (24%)	36 (28%)	0.267
**Prior Myocardial Infarction, n (%)**	21 (1.8%)	2 (1.6%)	>0.9
**Prior Atrial Fibrillation, n (%)**	139 (12%)	45 (35%)	<0.001
**Cancer, n (%)**	33 (2.9%)	2 (1.6%)	0.570
**Preprocedural LVEF (%)**	55.8 (14.7)	53.4 (15.5)	0.092
**Valve type, n (%)**			0.478
Self-expanding valve	925 (91%)	100 (90%)	
Balloon-expandable valve	75 (7.4%)	8 (7.2%)	
Mechanically expandable valve	14 (1.4%)	3 (2.7%)	
**LV, mm**	51 (46, 58)	50 (45, 59)	0.754

Characteristics	Cardiac damage Status<br>Early Stage (0~2)<br>N = 1,147	Advanced Stage (3~4)<br>N = 127	*P* value
**RV, mm**	42 (38, 47)	41 (37, 48)	0.568
**LA, mm**	22.00 (20.00, 23.00)	21.00 (20.00, 23.00)	0.439
**RA, mm**	36 (33, 40)	36 (33, 42)	0.371
**IVS, mm**	13.00 (12.00, 15.00)	13.00 (12.00, 14.00)	0.110
**LVPW, mm**	12.00 (10.00, 13.00)	12.00 (10.00, 12.00)	0.096

Abbreviations: The abbreviations are in accordance with Table S1.
