# Supplementary material for: Integrated Impact of Post-TAVR Cardiac Damage and Pacemaker Implantation on Long-Term Outcomes
Source: Biomedicines. 2026 Jul 13;14(7):1569. doi: 10.3390/biomedicines14071569 (PMC13406656; doi:10.3390/biomedicines14071569)
Supplement: Supplementary file 1 [file biomedicines-14-01569-s001.zip › Sup Table S1.pdf]

Table S1 Baseline Characteristics of Participants stratified by Postprocedural PPMI

Characteristics	Postprocedural PPMI<br>None PPMI<br>N = 1,050	 PPMI<br>N = 224	*P* value
**Age (y)**	73 (68, 78)	74 (69, 79)	0.012
**Gender, n (%)**			0.099
Male	598 (57%)	141 (63%)	
Female	452 (43%)	83 (37%)	
**BMI (kg/m²)**	22.8 (20.3, 25.1)	22.9 (20.9, 25.0)	0.482
**Hypertension, n (%)**	462 (44%)	100 (45%)	0.860
**Diabetes, n (%)**	217 (21%)	56 (25%)	0.151
**NYHA Class, n (%)**			0.555
1	11 (1.0%)	4 (1.8%)	
2	285 (27%)	63 (28%)	
3	610 (58%)	132 (59%)	
4	144 (14%)	25 (11%)	
**STS Score (%)**	3.2 (2.3, 6.2)	3.4 (2.1, 6.3)	0.783
**Creatinine Clearance (mL/min)**	53 (41, 67)	48 (36, 62)	0.001
**eGFR (mL/min/1.73 m²)**	69 (55, 86)	64 (49, 79)	0.007
**Chronic Obstructive Pulmonary Disease, n (%)**	258 (25%)	64 (29%)	0.211
**Cardiovascular Disease, n (%)**	168 (16%)	29 (13%)	0.251
**Chronic Kidney Disease, n (%)**	54 (5.1%)	21 (9.4%)	0.015
**Peripheral Vascular Disease, n (%)**	168 (16%)	36 (16%)	0.979
**Dialysis, n (%)**	8 (0.8%)	5 (2.2%)	0.062
**Coronary Artery Disease, n (%)**	254 (24%)	56 (25%)	0.798
**Prior Myocardial Infarction, n (%)**	19 (1.8%)	4 (1.8%)	>0.9
**Prior Atrial Fibrillation, n (%)**	148 (14%)	36 (16%)	0.445
**Cancer, n (%)**	28 (2.7%)	7 (3.1%)	0.703
**Preprocedural LVEF (%)**	55.5 (14.8)	56.1 (14.7)	0.473
**Valve type, n (%)**			0.370
Self-expanding valve	842 (91%)	183 (91%)	
Balloon-expandable valve	70 (7.6%)	13 (6.5%)	
Mechanically expandable valve	12 (1.3%)	5 (2.5%)	
**LV, mm**	51 (46, 58)	50 (45, 59)	0.754
**RV, mm**	42 (38, 47)	41 (37, 48)	0.568

	Postprocedural PPMI		
**Characteristics**	**None PPMI**<br>N = 1,050	**PPMI**<br>N = 224	***P* value**
**LA, mm**	22.00 (20.00, 23.00)	21.00 (20.00, 23.00)	0.439
**RA, mm**	36 (33, 40)	36 (33, 42)	0.371
**IVS, mm**	13.00 (12.00, 15.00)	13.00 (12.00, 14.00)	0.110
**LVPW, mm**	12.00 (10.00, 13.00)	12.00 (10.00, 12.00)	0.096

Abbreviations: PPMI: Permanent Pacemaker Implantation; NYHA: New York Heart Association; STS: Society of Thoracic Surgeons; eGFR: Estimated Glomerular Filtration Rate; LVEF: Left Ventricular Ejection Fraction; LV: Left Ventricle; RV: Right Ventricle; LA: Left Atrium; RA: Right Atrium; IVS: Interventricular Septum; LVPW: Left Ventricular Posterior Wall
