# Supplementary figures and images for: Integrated Impact of Post-TAVR Cardiac Damage and Pacemaker Implantation on Long-Term Outcomes
Source: Biomedicines. 2026 Jul 13;14(7):1569. doi: 10.3390/biomedicines14071569 (PMC13406656; doi:10.3390/biomedicines14071569)

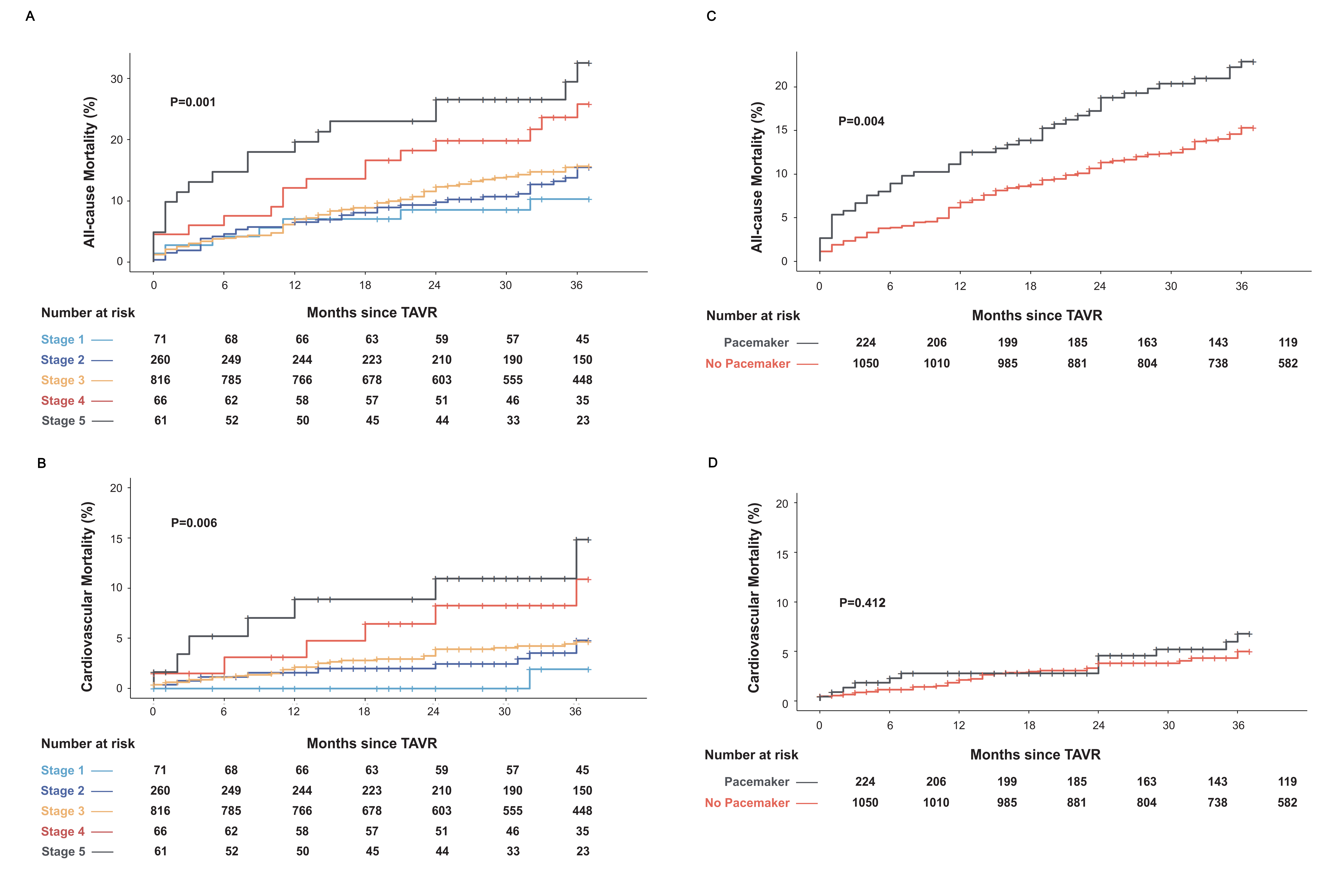

Supplement: Supplementary file 1 [file biomedicines-14-01569-s001.zip › Figure S1_01(2).png]
